# Effects of Acute Alcohol Intoxication on Empathic Neural Responses for Pain

**DOI:** 10.3389/fnhum.2017.00640

**Published:** 2018-01-04

**Authors:** Yang Hu, Zhuoya Cui, Mingxia Fan, Yilai Pei, Zhaoxin Wang

**Affiliations:** ^1^Shanghai Key Laboratory of Brain Functional Genomics, Key Laboratory of Brain Functional Genomics, Ministry of Education, Institute of Cognitive Neuroscience, Shanghai, China; ^2^Shanghai Key Laboratory of MRI, East China Normal University, Shanghai, China

**Keywords:** alcohol intoxication, pain empathy, fMRI, PPI, trait empathy

## Abstract

The questions whether and how empathy for pain can be modulated by acute alcohol intoxication in the non-dependent population remain unanswered. To address these questions, a double-blind, placebo-controlled, within-subject study design was adopted in this study, in which healthy social drinkers were asked to complete a pain-judgment task using pictures depicting others' body parts in painful or non-painful situations during fMRI scanning, either under the influence of alcohol intoxication or placebo conditions. Empathic neural activity for pain was reduced by alcohol intoxication only in the dorsal anterior cingulate cortex (dACC). More interestingly, we observed that empathic neural activity for pain in the right anterior insula (rAI) was significantly correlated with trait empathy only after alcohol intoxication, along with impaired functional connectivity between the rAI and the fronto-parietal attention network. Our results reveal that alcohol intoxication not only inhibits empathic neural responses for pain but also leads to trait empathy inflation, possibly via impaired top-down attentional control. These findings help to explain the neural mechanism underlying alcohol-related social problems.

## Introduction

Alcohol is favored for its acute ability to induce positive affect by activating neural reward systems (Fromme et al., 1999), as well as reduce stress and anxiety (Curtin and Lang, 2007). However, alcohol consumption, especially at high doses, is also associated with severely impaired social abilities, such as more aggressive behavior (Bushman and Cooper, 1990). Denson et al. (2008) has hypothesized that alcohol might increase aggression via a reduction in the ability to identify with and vicariously share the feeling, pain and thoughts of others, i.e., empathy (Denson et al., 2008), as the level of empathy is negatively correlated with the intensity and frequency of violence in sobriety (Miller and Eisenberg, 1988). Empathy is believed to be a key motivator and the proximate mechanism of altruistic and prosocial behavior (Preston and de Waal, 2002; Singer et al., 2006; Moriguchi et al., 2007). Thus, any change in empathy by alcohol consumption will have social implications.

In recent years considerable efforts have been made to investigate the neural correlates of human empathy. The majority of studies used experimental paradigms in which participants were exposed to stimuli depicting or indicating that other people were in pain. Recent imaging studies revealed that empathy for pain recruits a distributed network, including the anterior cingulate cortex (ACC) and bilateral anterior insula (AI), major components of self-pain related “affective pain matrix” (Jackson et al., 2005; Hein and Singer, 2008; Singer and Lamm, 2009). Alcohol acts mainly as a central nervous system depressant. Previous neuroimaging studies indicated that alcohol dampened activity in the dorsal anterior cingulate cortex (dACC) (Ridderinkhof et al., 2002; Marinkovic et al., 2012) and the bilateral anterior insula (AI) (Padula et al., 2011) in tasks other than empathy. As these three regions that were affected by alcohol consumption are considered the core affective pain matrix of empathy (Lamm et al., 2010a; Fan et al., 2011), we hypothesized that empathic responses for pain in these regions may be reduced, and the three regions that affected by alcohol intoxication (dACC, bilateral AIs) were taken as regions of interest (ROI).

The second question we want to address is whether the effects of trait empathy on the empathic neural responses are altered by alcohol consumption. Interestingly, the majority of empathy studies with sober participants, if not all, have not found any correlation between trait empathy and the activity of the affective pain matrix (Decety, 2011). However, Giancola (2003) demonstrated that trait empathy, the tendency for people to imagine and experience the feelings and experiences of others, has moderating effects on alcohol-related aggression in men and women but not in men and women in sobriety (Giancola, 2003). These results suggest that the effects of trait empathy on behavior was affected by alcohol intoxication, as well as underlying neural correlates.

More important, attention plays an important role in empathy. Previous studies revealed that activity in the affective pain matrix is reduced, even absent, if attention was diverted away (Gu et al., 2010; Lamm et al., 2010b). Thus, activity in the affective pain matrix may be more affected by trait empathy if attention is impaired. The capacity to divide and sustain attention is indeed impaired by alcohol, even at blood-alcohol concentration (BAC) levels of 0.02–0.03% (Koelega, 1995). Therefore, in accordance with Giancola's finding, we predicted empathic neural responses were more affected by trait empathy in the intoxication condition instead of the placebo condition. Our results confirmed this prediction and found that brain activity in the rAI was correlated with trait empathy only in the alcohol condition.

Another intriguing point is that as the affective pain matrix, but not a single brain region, is involved in empathic processing of pain, there are likely interactions between these different brain regions. Noted that functional connection between brain regions that are engaged simultaneously in a task (Rogers et al., 2008) can be affected by task-related parameters (Friston et al., 1997), i.e., psychophysiological interactions (PPI). For example, Friston KJ et al provided an example of physiological interactions in the visual pathway were modulated by attention (Friston et al., 1997). As attention was also impaired by alcohol consumption (Steele and Josephs, 1990; Ridderinkhof et al., 2002; Calhoun et al., 2004; Van Horn et al., 2006; Marinkovic et al., 2012) and that empathic responses for pain were modulated by attention (Gu and Han, 2007), the functional connection between brain regions could be affected by alcohol consumption. To test this hypothesis, we further conducted a psychophysiological interactions (PPI) analysis (Friston et al., 1997) with rAI as the seed in both alcohol and placebo conditions.

## Methods

### Participants

Twenty-one native Chinese students from East China Normal University (13 males; age = 23.4 ± 2.0 years old) volunteers attended the current study, 16 of them were available for final data analysis (10 males; age = 23.4 ± 2.1 years old). All participants were light or moderate social drinkers (once a week or less, with low quantity and no binging) (Lipton, 1994) and had scores on the Short Michigan Alcoholism Screening Test (SMAST) of <5 (Selzer et al., 1975). Other inclusion criteria were as follows: (1) right-handed with normal or corrected-to-normal vision, normal color perception; (2) no self-reported history of psychiatric or neurological disease, head injury, or drug abuse, and scores on the Beck Depression Index (BDI) were <13 (Beck et al., 1961); and (3) not allergic to orange juice. The participants were paid as compensation for their time. Written informed consent were obtained from all subjects, and the protocol was approved by the University committee on Human Research Protection (UCHRP) at East China Normal University. A power calculation based on the effect size of dACC (Hedges' unbiased d = 0.63 for left dACC and 0.56 for right dACC) reported in a previous study (Marinkovic et al., 2012) was performed using G^*^Power (Faul et al., 2009), resulting in about 12–16 participants with the power of 0.70. Note that in Marinkovic et al.'s study, the neural basis of alcohol's effects on cognitive control was investigated using a Siemens 3T scanner, we expected the alcohol's effects on the same brain regions using the same type of scanner and the same event-related design be similar.

### Materials

#### Stimuli

**Figure 1:**
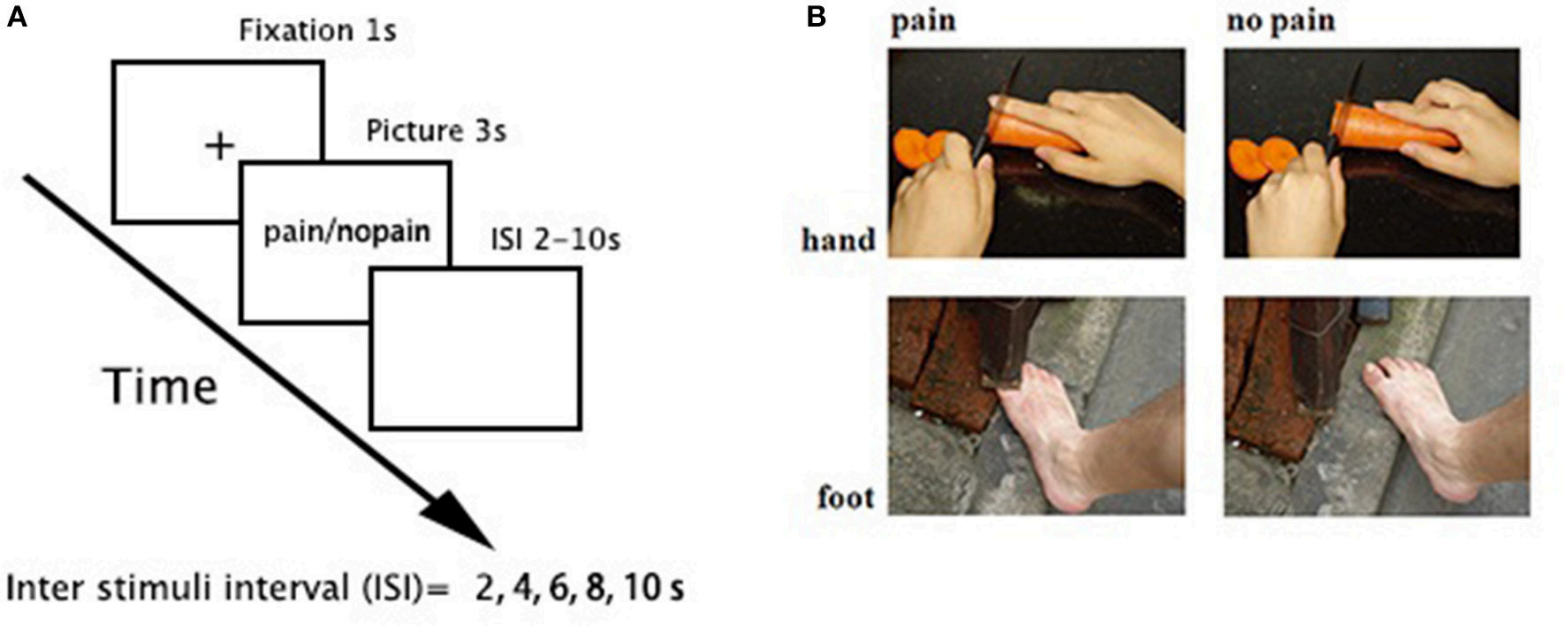
Task paradigm. **(A)** a 3-s picture was presented in each trial, preceded by 1-s fixation and followed by a pseudorandom jittered inter stimuli interval (ISI, 2, 4, 6, 8, and 10 s, with a mean = 6 ± 2 s). The order of stimuli was pseudorandomly mixed in each run. **(B)** sample stimuli.

One-hundred and sixty digital color pictures depicting right hands/feet in painful and non-painful everyday situations (80 each) from first-person perspective were used (e.g., Figure 1). The pictures were equally divided into two sets (each set consisted of 40 painful/non-painful pictures), one for the placebo condition and the other for the alcohol condition, and counterbalanced among participants. All pictures were presented twice to increase the signal-to-noise ratio. Among all stimuli, 104 pictures (52 painful pictures) were courtesy of Jackson and Decety (Jackson et al., 2005), and we created another 56 images (28 painful pictures) using a similar style. All pictures were resized to the same resolution (600 × 480 pixels).

#### Beverage

Two types of beverages were used, containing either alcohol (0.85 g/kg, alcohol condition) or water (placebo condition) (Ridderinkhof et al., 2002). Body-weight dependent measures of ErGuoTou (one of the China's favorite liquor with a relatively high alcohol percentage, 55 or 56%) or an equal volume of water were dissolved in orange juice such that the total liquid volume was 400 mL. A sip of ErGuoTou (about 1 mL, <0.015g/kg) was also added to the placebo condition as a taste mask.

### Procedure

A double-blind, placebo-controlled, within-subject design was used. There were two sessions scheduled at least 7 days apart, one for the alcohol condition and the other for the placebo condition. The order of the two sessions was counterbalanced between participants. The beverages were prepared by one of the authors (Z.X.W.). Neither the experimenters (Y.H., Z.Y.C., and M.X.F.) nor the data analyst (Y.H.) knew the beverage contents.

All participants were instructed to abstain from alcoholic beverages and recreational/psychoactive drugs for at least 48 h prior to each session, and were asked to eat only a light meal (avoiding fatty foods) 40–60 min prior to each fMRI session.

During each session, the participants first filled out the Interpersonal Reaction Index (IRI), an empathy trait scale (Davis, 1983), upon arrival. Then, they were asked to consume the beverage within 20–25 min. After a 25-min rest, their affect states were measured by the Positive Affect and Negative Affect Scale (PANAS) (Watson et al., 1988). Then, the participants were positioned in the scanner with foam padding around the head to minimize motion. The functional MRI runs commenced approximately 55 min after beverage ingestion, timed such that the upcoming cognitive task would concur with peak blood alcohol levels (Sripada et al., 2011).

An event-related fMRI design was applied (Figure 1). There were four functional runs. Each run consisted of 40 trials (20 painful). In each trial, a 3-s picture was presented, preceded by 1-s fixation and followed by a pseudorandom jittered inter stimuli interval (ISI, 2, 4, 6, 8, and 10 s, with a mean = 6 ± 2 s). The order of stimuli was pseudorandomly mixed in each run. Participants were instructed to judge whether the model in the stimuli was painful or not and responded with their right hand using a hand-shaped response box.

After scanning, the participants were again presented each picture and required to rate (1) the pain intensity felt by the model and the (2) subjective unpleasantness while watching, both on a 9-point Likert scale (1 = no pain/no unpleasantness, 9 = very painful/very unpleasant). Then, the participants were offered to be escorted back to their dorms and were advised to have a rest.

### Image acquisition

The scanning was conducted using a 3-Tesla Siemens Trio MR scanner using a 12-channel head coil and included 4 functional runs and 1 anatomical run. For functional images, 35 axial slices (FOV = 240 × 240 mm^2^, matrix = 64 × 64, in-plane resolution = 3.75 × 3.75 mm^2^, thickness = 4 mm, without gap) covering the whole brain were obtained using a T2^*^-weighted echo planar imaging (EPI) sequences (TR = 2,000 ms, TE = 30 ms, flip angle = 90°). A high-resolution structural image for each participant was acquired using 3D MRI sequences for anatomical co-registration and normalization (TR = 1,900 ms, TE = 3.43 ms, flip angle = 7°, matrix = 256 × 256, FOV = 240 × 240 mm^2^, slice thickness = 1 mm, without gap).

### Data analysis

#### Whole-brain analysis

Data from five participants was excluded because of poor behavioral performance (accuracy < 50%; *n* = 2), disruption of the inebriation procedure (*n* = 2), or quitting (*n* = 1). SPM8 was adopted for data analysis (Wellcome Department of Cognitive Neurology, London, UK; http://www.fil.ion.ucl.ac.uk/spm/). For each session of each participant, the EPI images were first realigned to the first volume to correct for head motions. Then, the anatomical image was co-registered with the mean EPI image, segmented and then generated normalized parameters to MNI space. Using these parameters, all EPI data were projected onto MNI space with a 2 × 2 × 2 mm^3^ resolution and then smoothed using an 8-mm FWHM (full width half maximum) isotropic Gaussian kernel. High-pass temporal filtering with a cut-off of 128 s was performed to remove low-frequency drifts.

For the first level analysis, a general linear model with two conditions (i.e., “pain” and “nopain”) convolved with the canonical hemodynamic response function (HRF) was applied. The six estimated head movement parameters were included in the design matrix to remove the residual effects of head motion. Parameter estimates were then entered into the second level analysis using a 2 × 2 repeated measures ANOVA for exploration purpose, with stimuli type (painful vs. non-painful) and beverage type (alcohol vs. placebo) as independent factors. A voxel-wise threshold was set at *p* < 0.001 (*k* = 40) was adopted.

#### ROI analysis

Note that in pharmacological fMRI, prior hypotheses in functional brain imaging are often formulated by constraining the data analysis to ROIs, and this approach yields higher sensitivity than whole brain analyses (Mitsis et al., 2008). Thus, an ROI approach was performed to determine whether empathic responses for pain were reduced by alcohol intoxication using the AFNI software package (Cox, 1996). Three core regions of empathy were defined as ROIs, e.g., the dACC and bilateral AIs, with the following MNI (Montreal Neurological Institute) coordinates, adopted from a recent meta-analysis (Fan et al., 2011): dACC, x/y/z = −2/24/38; lAI/IFG, x/y/z = −42/18/0; and rAI/IFG, x/y/z = 38/24/−2. All ROIs were set as a sphere with a radius of 6 mm. For each ROI, the parameter estimates of 16 participants were extracted for further repeated measures ANOVA analysis and a paired *t*-test as post hoc analysis, with the threshold set to *p* = 0.05 (two tailed).

#### Regression analysis

A regression analysis was further applied to determine whether the empathic neural responses (i.e., pain > nopain) were mediated by trait empathy, using the following regressors: (1) the Empathic Concern subscale of the IRI (IRI-EC), i.e., the index of trait empathy, (2) mean behavioral index of pain intensity (contrast of pain vs. nopain), and (3) mean subjective rating of unpleasantness (contrast of pain vs. nopain), both in the placebo and alcohol conditions. A voxel-wise threshold was set at *p* < 0.001 within an inclusive mask (pain > nopain; p < 0.05, uncorrected; *k* = 100) was adopted and all brain regions survived FDR correction at cluster level.

#### Psycho-physiological interaction (PPI) analysis

The regression analysis revealed that activity in the rAI was modulated by trait empathy only in the intoxicated participants. A further PPI analysis (Friston et al., 1997) was then performed, with the rAI as seed. First, the source mask was defined as two 8-mm spheres centered at the peak voxel in the rAI (x/y/z = 40/16/−4) from main effect of empathy. The seed volume of interest (VOI) for each individual was then defined as a sphere with a 6-mm-radius centered at the peak voxel from the contrast of pain > nopain within these masks. The time series of each VOI was then extracted, and the PPI regressor was calculated as the element-by-element product of the mean-corrected activity of this VOI (the physiological regressor) and a vector coding for the differential stimuli effects of pain > nopain (the psychological regressor). These regressors were convolved with the canonical HRF and then entered into the regression model along with six head motion parameters. The individual contrast images were subsequently subjected to one-sample *t*-tests. Lastly, group analysis was applied to identify the brain regions displayed increased functional connectivity with the seed VOI (i.e., rAIs) during pain empathy both in the placebo and alcohol conditions. A voxel-wised threshold was set at *p* < 0.001 (uncorrected) and all brain regions survived FDR correction at cluster level.

## Results

### Dispositional measures

All scores of the dispositional measures were in the normal range (Table 1). The participants rated slightly higher in the positive affect after drinking in comparison with that in the placebo condition [PA-placebo: mean = 2.8 (*SD* = 0.8); PA-alcohol: 3.1(0.7); *t*_(15)_ = 2.17, *p* = 0.046], but not in negative affect [NA-placebo: 1.3(0.4); NA-alcohol: 1.4(0.4); *t*_(15)_ = 1.16, *p* = 0.263].

**Table 1:** Scores on trait scales (*N* = 16).

**Scale**	**Scores (*SD*)**
Interpersonal Reaction Index (IRI)	64.2 (10.6)
Empathic concern subscale (EC)	18.9 (3.1)
Fantasy subscale (FA)	15.7 (4.8)
Perspective-taking subscale (PT)	17.6 (3.2)
Personal distress subscale (PD)	12.1 (3.4)
Short Michigan Alcoholism Screening Test (SMAST)	0.4 (0.6)
Beck Depression Inventory (BDI)	4.1 (3.2)

### Behavioral results

As for reaction time (RT), both a main effect of the beverage and stimuli type was detected [*F*s _(1, 13)_ > 8.1, *p*s < 0.014, η^2^ > 0.38], and the participants responded slower toward painful stimuli (v.s. non-painful stimuli) in both conditions [*t*s _(13)_ > 2.2, *p*s < 0.045] and that they displayed longer RT in the alcohol (vs. placebo) condition regardless of stimuli [*t*s _(13)_ > 2.6, *p*s < 0.023]. Neither a main effect nor a stimuli type×beverage interaction was found in accuracy [*F*s _(1, 13)_ < 4.2, *p*s > 0.06, η^2^ < 0.23]. Note behavioral data from two participants during scanning were lost because of technical reasons.

As for the post-scanning rating scores, a main effect of stimuli type was found in both pain intensity and unpleasantness [*F*_(1, 15)_ > 97.0, *p* < 0.001, η^2^ > 0.8], whereas an interaction effect was only detected in unpleasantness [*F*_(1, 15)_ = 4.7, *p* = 0.046, η^2^ = 0.24]. The participants rated significantly higher on painful (vs. non-painful) stimuli regardless of beverage in both pain intensity [*t*s_(15)_ > 15.3, *p* < 0.001] and unpleasantness [*t*s_(15)_ > 9.3, *p* < 0.001]. Moreover, the unpleasantness rating difference between the painful stimuli and non-painful stimuli in the alcohol condition was larger than that in the placebo condition [*t*_(15)_ = 2.18, *p* = 0.046]. The average values of these behavioral measurements are listed in Table 2.

**Table 2:** Average behavioral performance during and after fMRI scanning.

**Measurements**	**Condition**	**Accuracy (%;** ***SD*****)**			**RT (ms;** ***SD*****)**		
		**Painful**	**Non-painful**	**Total**	**Painful**	**Non-painful**	**Total**
During Scanning	Placebo	88.7 (7.6)	93.8 (5.4)	91.2 (7.0)	1284 (279)	1190.0 (235)	1237.1 (257)
(*N* = 14)	Alcohol	89.8 (6.5)	91.4 (6.2)	90.6 (6.3)	1421 (192)	1299.8 (198)	1360.3 (201)
		**Intensity (*****SD*****)**			**Unpleasantness (*****SD*****)**		
		**Painful**	**Non-painful**	**Total**	**Painful**	**Non-painful**	**Total**
After Scanning	Placebo	7.5 (0.9)	1.6 (0.3)	4.5 (0.9)	7.8 (0.6)	4.0 (1.5)	5.9 (0.8)
(*N* = 16)	Alcohol	7.5 (1.0)	1.7 (0.3)	4.6 (0.8)	7.9 (0.8)	3.6 (1.6)	4.7 (0.9)

*Behavioral data from 2 participants during scanning were lost because of technical reasons*.

### Imaging results

#### Whole-brain results

**Figure 2:**
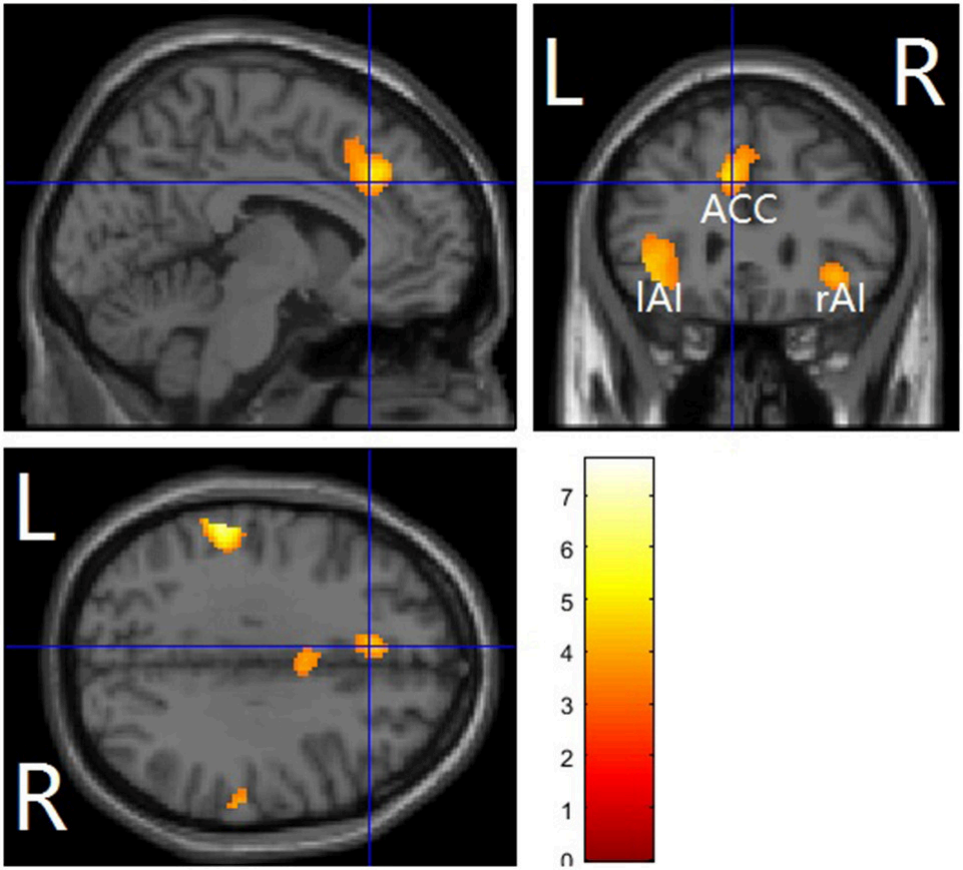
Pain vs. nopain across sobriety /intoxication in a whole-brain analysis. The affective pain matrix was displayed. Voxel-wised threshold was set at *p* = 0.001, FDR corrected at cluster-level. L, left; R, right; ACC, anterior cingulate cortex/supplementary motor area; AI, anterior insula.

**Figure 3:**
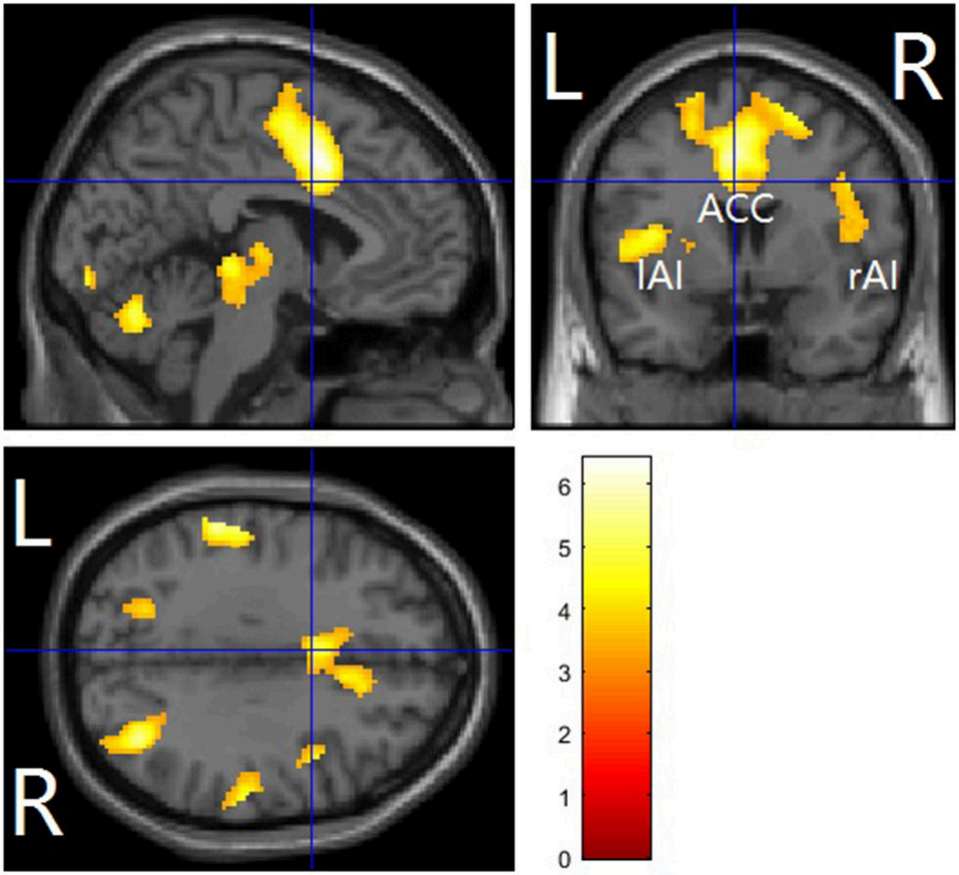
Alcohol vs. placebo across pain and nopain conditions in a whole-brain analysis. The affective pain matrix was displayed. Voxel-wised threshold was set at *p* = 0.001. FDR corrected at cluster-level. L, left; R, right; ACC, anterior cingulate cortex/supplementary motor area; AI, anterior insula.

The main effects of empathy for pain (i.e., painful > non-painful) were found in the ACC/SMA (BA 6/8/24/32), bilateral AIs (BA 13) extending to IFG (BA 47), bilateral inferior parietal lobule (IPL, BA 7/39/40) and bilateral somatosensory cortex (BA 1/2/3), see Table 3 and Figure 2 for details. The beverage main effect (placebo > alcohol) was found in the mid-cingulate/SMA (BA 6/24), bilateral IPL extending to the supra-marginal gyrus (BA 7/40), left mid-insula (BA 13), and bilateral visual cortices (BA 19/37), along with several sub-cortical areas, including the hippocampus and thalamus, see Table 4 and Figure 3 for details. No significant interaction was found.

**Table 3:** Main effects of empathy for pain.

**Contrasts**	**Regions**	**Side**	**Brodmann area (BA)**	**MNI coordinates**			**Cluster size**	**T score**
				**x**	**y**	**z**		
Pain > nopain	dACC/MeFG	L/R	9/32	−6	31	32	407	4.92^**^
	AI/IFG	L	13/47	−40	19	−1	1,330	5.55^***^
		R	13/47	36	29	−6	257	4.21^*^
	IPL	L	7/40	−55	−31	37	1,089	7.68^***^
Nopain > pain	MFG	L	8	−26	27	35	406	4.32^**^
		R	8	26	29	39	395	7.32^**^
	IPL/AG	L	7/39	−40	−68	44	1,193	6.93^***^
	SMG/AG	R	39/40	55	−55	30	2,034	6.63^***^
	MTG	L	21	−53	0	−8	632	6.39^***^
		R	21	59	1	−15	337	5.98^*^
	Precuneus/LG	L/R	7/19	4	−54	41	6,352	6.16^***^
	ACC	R	32	18	45	7	244	5.8^*^

L, left; R, right; dACC, dorsal anterior cingulate cortex; SMA, supplementary motor area; MeFG, medial frontal gyrus; CG, cingulated gyrus; MFG, middle frontal gyrus; IFG, inferior frontal gyrus; AI, anterior insula; PI, posterior insula; PCG, pre-central gyrus; PoCG, post-central gyrus; SMG, supramarginal gyrus; IPL, inferior parietal gyrus; AG, angular gyrus; MTG, middle temporal gyrus; LG, lingual gyrus;

* *p < 0.05*,

** *p < 0.01*,

*** *p < 0.001, FDR corrected at cluster-level*.

**Table 4:** Main effects of beverage.

**Contrasts**	**Regions**	**Side**	**Brodmann area (BA)**	**MNI coordinates**			**Cluster size**	**T score**
				**x**	**y**	**z**		
Placebo > alcohol	SMA/MCC/PCG	L/R	6/24	−6	10	38	2766	6.41^***^
	IPL/PoCG/Precuneus	L	2/40	−59	−32	27	2079	5.9^***^
	Insula	L	13	−40	0	9	346	5.45^**^
	Midbrain/Thalamus	L		−8	−17	1	512	5.43^***^
	Thalamus	R		10	−25	0	593	5.06^***^
Alcohol > placebo	AG/IPL	L	39	−51	−60	42	256	4.75^*^

*L, left; R, right; SMA, supplementary motor area; MCC, mid-cingulate gyrus; IFG, inferior frontal gyrus; PCG, pre-central gyrus; PoCG, post-central gyrus; IPL, inferior parietal gyrus; AG, angular gyrus; FG, fusiform gyrus; MOG, middle occipital gyrus; PhG, parahippocampal gyrus*;

* *p < 0.05*,

** *p < 0.01*,

*** *p < 0.001, FDR corrected at cluster-level*.

#### ROI results

**Figure 4:**
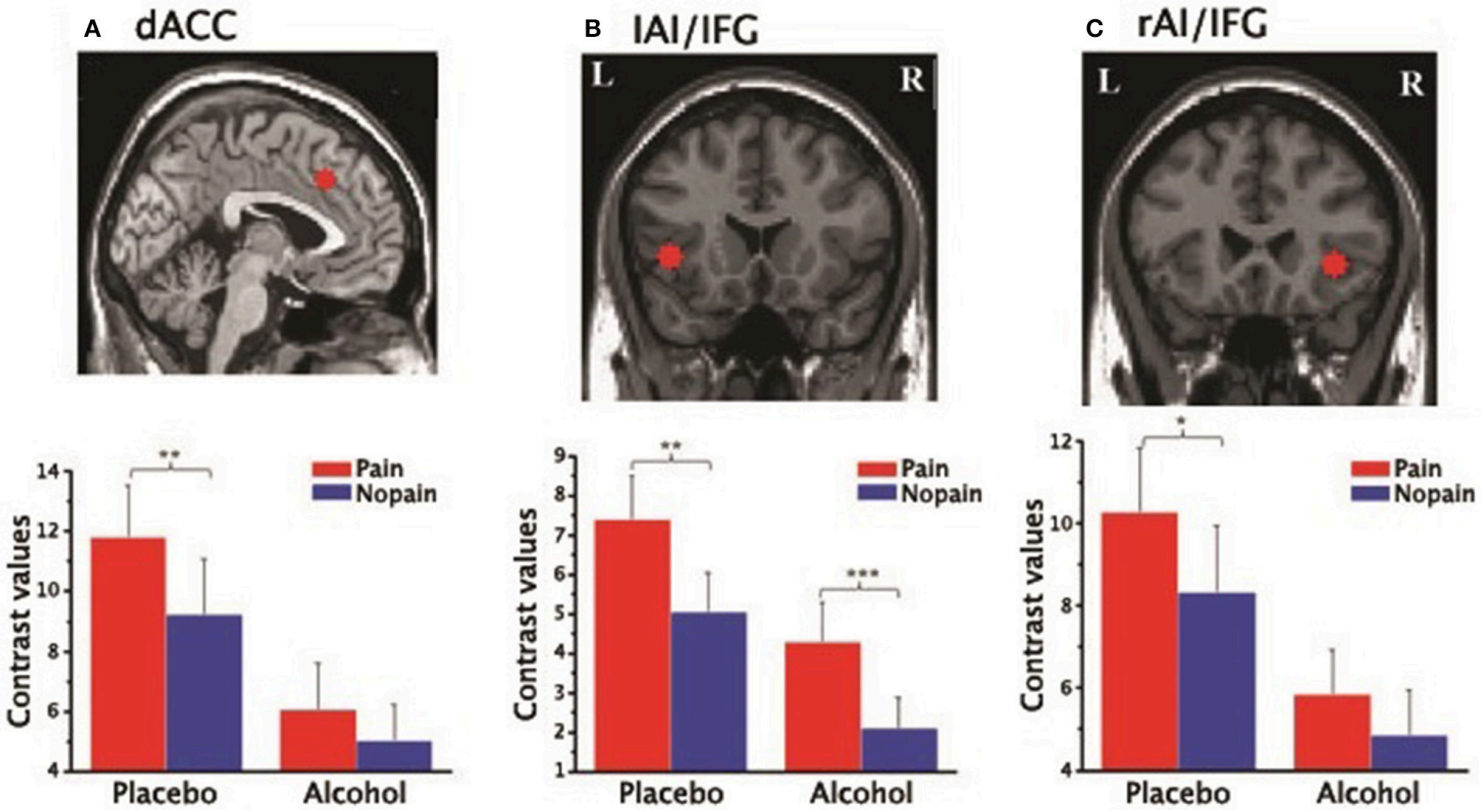
Contrast values of the parameter estimates of the three ROIs of the affective pain matrix in the placebo and alcohol condition: **(A)** results for the dorsal anterior cingulate cortex (dACC); **(B)** results for the left anterior insula extending to the inferior frontal gyrus (lAI/IFG); **(C)** results for the right anterior insula extending to the inferior frontal gyrus (rAI/IFG). L, left; R, right; ^*^p < 0.05, ^**^p < 0.01, ^***^p < 0.001; The error bars represent the SEM.

In the three ROIs of the affective pain matrix, both main effects of stimuli type and beverage were observed [*F*s_(1, 15)_ > 6.52, *p*s < 0.022, η^2^ > 0.3; Figure 4]. However, a significant interaction was found only in the dACC [*F*_(1, 15)_ = 4.99, *p* = 0.041, η^2^ = 0.24]. A post-hoc paired *t*-test indicated that increased empathic activity for pain (i.e., pain > nopain) in the dACC along with the rAI/IFG was found in the placebo condition (*t*s_(15)_ > 2.71, *p*s < 0.016] but not in the alcohol condition [*t*s_(15)_ < 2.11, *p*s > 0.053], whereas the left AI/IFG displayed enhanced activation from painful (vs. non-painful) stimuli regardless of the beverage type [*t*s_(15)_ > 3.08, *p*s < 0.008]. In addition, all ROIs displayed reduced activity to both types of stimuli after alcohol intoxication compared with the placebo condition [*t*s_(15)_ < −2.17, *p*s < 0.045]. No other main effect or interaction was detected.

#### Regression results

**Figure 5:**
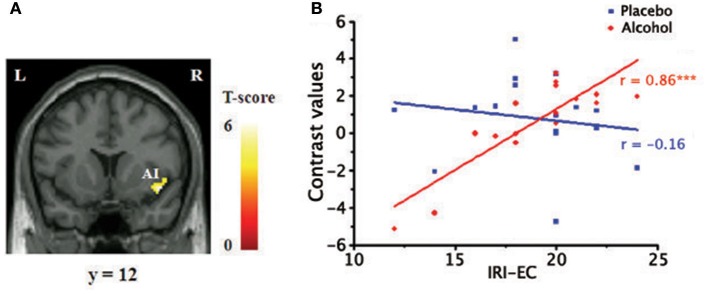
Positive relationship between empathy concern subscale of Interpersonal Reaction Index (IRI-EC) scores and empathic neural responses for pain in the right AI. **(A)** A significant cluster superimposed onto a coronal section (*p* < 0.001, FDR corrected at cluster level), masked by the main effect of empathy (pain > nopain; p < 0.05); **(B)** A scatter plot of the positive correlation in the peak voxel for display purposes (x/y/z=44/12/-12, MNI coordinates). ^***^p < 0.001.

Within the core affective pain matrix, the voxel-wised regression analysis revealed that empathic activity for pain in the rAI was significantly positively related with the scores of the IRI-EC in the alcohol condition (Figure 5A) when the pain intensity and unpleasant levels were controlled. The plot of the neural activity at the peak voxel vs. the score was displayed in Figure 5B (Pearson's correlation, *r* = 0.86, *p* < 0.001).

#### PPI results

**Figure 6:**
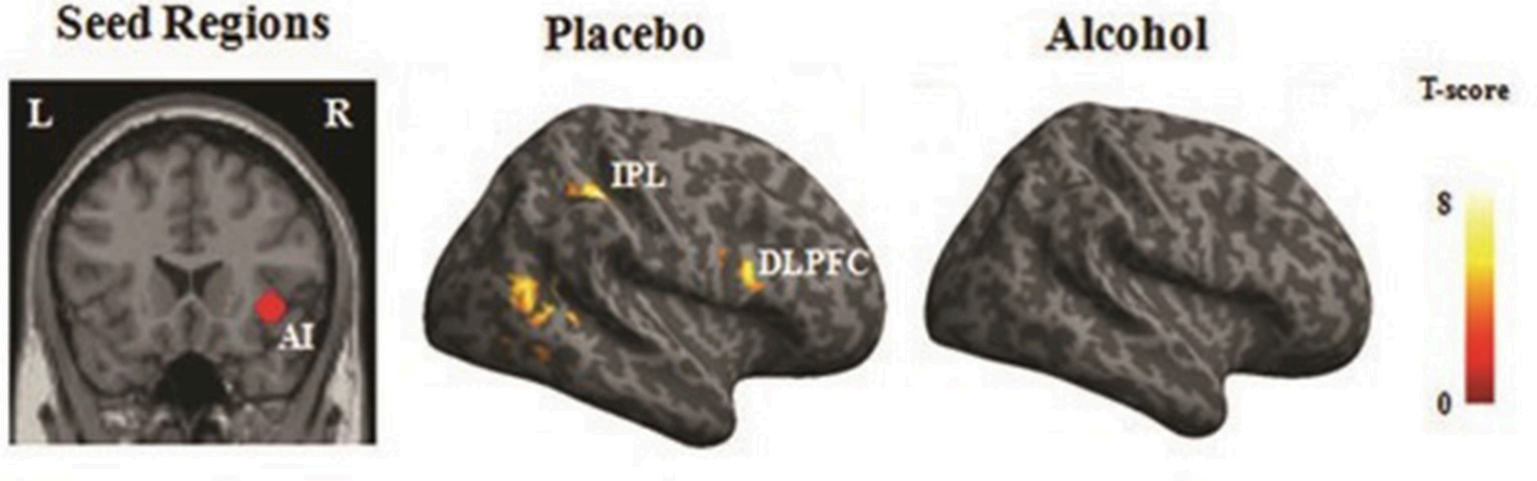
PPI results. Regions that displayed increased functional connectivity with the right AI (rAI) during empathy for pain in the placebo and alcohol condition (*p* < 0.001). The right hemisphere is displayed. L, left; R, right; AI, anterior insula; DLPFC, dorsal lateral prefrontal cortex; IPL, inferior parietal gyrus.

Significant functional connectivity was observed between the rAI and the fronto-parietal areas, including the right dorso-lateral prefrontal cortex (BA 44) along with the bilateral IPL (BA 39), as well as the visual cortices during empathy for pain (i.e., pain > nopain) in the placebo condition but not in the alcohol condition (Figure 6), see Table 5 for details.

**Table 5:** Increased PPIs of the seed region during empathy for pain in the placebo and alcohol conditions (*p* < 0.001; *k* = 100).

**Condition**	**Seed region**	**Regions**	**Side**	**Brodmann area (BA)**	**MNI coordinates**			***k***	**T score**
					**x**	**y**	**z**		
Placebo	rAI	SMA	L/R	6	4	6	52	101	5.5^*^
		IPL	L	40	−42	−42	44	410	6.67^**^
			R	39	40	−35	44	229	8.21^**^
		PhG/MOG	L	19/37	−32	−51	−4	189	6.07^**^
		DLPFC	R	44	48	13	23	212	7.41^**^
		STG/MTG	R	22	48	−57	18	373	8.14^***^
		FG	R	20	40	−40	−13	159	5.8^*^
Alcohol	rAI	FG	R	37	26	−38	−15	261	7.77^***^

*rAI, right anterior insula; SMA, supplementary motor area; DLPFC, dorso-lateral prefrontal cortex; IPL, inferior parietal gyrus; PhG, parahippocampal gyrus; TPJ, temporo-parietal junction; STG, superior temporal gyrus; MTG, middle temporal gyrus; FG, fusiform gyrus; LG, lingual gyrus; MOG, middle occipital gyrus*;

* *p < 0.05*,

** *p < 0.01*,

*** *p < 0.001, FDR corrected at cluster-level*.

## Discussion

The present study was carried out to investigate the effects of alcohol consumption on empathy for pain. A significant interaction was found in the dACC. As the dACC is considered to be one of the core regions of the pain empathy-related network (Lamm et al., 2010a) to code the value of affective stimulus (Fan et al., 2011), our results suggest that the perceived affective value of empathic concern was impaired by alcohol intoxication (Steele and Josephs, 1990), which is in line with our hypothesis. Note that the dACC also plays a key and necessary role in voluntary and cognitive control (Shackman et al., 2011), such as resolving conflicts (Botvinick et al., 2001) or monitoring errors (Yeung et al., 2004; Marinkovic et al., 2012). Thus, reduced perceived affective value should result in an underestimation of others' pain as well as poor cognitive control, and then lead to poor control of aggressive behaviors (Steele and Josephs, 1990).

One of the most interesting findings was that the empathic responses in the rAI were positively correlated with trait empathy in the alcohol condition, but not in the placebo condition. This finding is not due to changes of affective states or behavioral responses after drinking, as these confounding factors are ruled out in the regression analysis. The insula has long been considered one of the key regions that related with traits. For example, activation in insula was also found closely related to various trait measures, such as anxiety (Simmons et al., 2006) or intolerance (Simmons et al., 2008). Thus, our results indicate insula is also related with trait empathy, and intoxication leads to trait empathy inflation. These findings significantly extends the self-inflating phenomenon (i.e., enhancing feelings of self-appraisal) (Steele and Josephs, 1990) to the social cognition domain of trait empathy in intoxication.

Moreover, we found that the functional connectivity between the rAI and attention network, which occurred during empathy for pain in the placebo condition, was missing in the alcohol condition. It is believed that attention plays an important role in empathy. For example, empathic responses in the AIs were significantly reduced when the participants were distracted (Gu and Han, 2007) or cognitively busy (Rameson et al., 2011). AIs activity during a pain perception task was also significantly reduced when higher activity was observed in the right dorsolateral prefrontal cortex (Lorenz et al., 2003). Considering that activities in the fronto-parietal attention/control network, including the SMA/ACC, bilateral dorsolateral frontal cortices (middle/inferior frontal gyri), and bilateral parietal areas, were significantly decreased by alcohol intoxication, we conclude that crosstalks between the rAI and attention network is impaired by alcohol consumption, resulting in impaired top-down modulation of the rAI.

Taken together, the most likely explanation is that empathy for pain is shaped by other factors (de Waal, 2007), such as social norm. Thus, people with both high and low trait empathy may display relatively normal empathy responses for pain in sobriety. However, as alcohol is a central nervous depressant, it seems that alcohol intoxication not only inhibits local neural activity, but also inhibits neural signal flow between brain regions. The communication between the attention network and rAI was downregulated, and influences of other factors on empathy were thus reduced, resulting in trait empathy inflation. These results provide a possible neural foundation for the finding that empathy plays a moderating role in alcohol-related aggression behaviors (Giancola, 2003).

Despite the fact that alcohol intoxication led to those neural changes, participants could still differentiate painful stimuli from non-painful stimuli correctly as well as their intensities after drinking. These findings not only undermine the possibility that participants were disengaged from our task after alcohol intoxication, they also suggest that there is disassociation between cognitive appraisal and neural empathic activation for pain after drinking. Preserved cognitive appraisal but impaired brain function in other domains such as affective information has been reported during alcohol intoxication (Padula et al., 2011). A recent lesion study revealed that only damage to the AIs rather than the dACC might be more necessary for affecting pain empathy and causing impaired behavioral empathic responses (Gu et al., 2012). Hence, the ability to correctly judge other's affective state may be preserved because of the relatively preserved empathic responses in the AIs. It is also possible that other factors (e.g., past knowledge) may be involved. These disassociations suggest that humans have an inability to acknowledge their social dysfunction after drinking (Fromme et al., 1999).

There are two limitations. The first one is that although the double-blindness procedure used in our manuscript is widely used in previous studies such as Ridderinkhof et al. (2002), it remains possible that participants became aware of the experimental condition given the strong behavioral effects of alcohol over water placebo and our results can be affected by participant awareness. However, we think that our results are robust as the observed significant correlation between empathic neural activity in the right anterior insula (rAI) and trait empathy cannot be interpreted by participant awareness. Second, our sample size is relatively small (data from 16 participants were valid). We also note that a significant stimuli type×beverage interaction was only found in the ROI analysis, but not in the voxel-wised whole brain analysis, and no interaction was found in the AIs, possibly due to the small sample size. These results are in line with a previous suggestion that an ROI approach yields higher sensitivity than whole brain analyses in pharmacological fMRI with prior hypotheses (Mitsis et al., 2008). Further studies using a larger sample size are desired.

In summary, we provide the first piece of evidence that the empathic neural response for pain in the dACC is impaired by acute alcohol consumption in a group of healthy social drinkers. Moreover, we also show that alcohol intoxication leads to inflated trait empathy in the rAI. The neural correlates underlying trait empathy inflation are likely due to the impaired crosstalk between the rAI and attention neural network, i.e., impaired top-down modulation. Whether similar mechanisms are involved in other alcohol-related self-inflation deserves further investigation.

## Author contributions

YH, MF, ZW, and ZC: designed the study; YH, ZC, and MF: collected the data; YH: analyzed the data; YH, YP, and ZC: wrote the paper.

### Conflict of interest statement

The authors declare that the research was conducted in the absence of any commercial or financial relationships that could be construed as a potential conflict of interest.
